# Total Flavonoids Extracted from* Oxytropis falcata* Bunge Improve Insulin Resistance through Regulation on the IKK*β*/NF-*κ*B Inflammatory Pathway

**DOI:** 10.1155/2017/2405124

**Published:** 2017-03-26

**Authors:** Lixia Yang, Zhicheng Wang, Liangen Jiang, Wen Sun, Qiang Fan, Tonghua Liu

**Affiliations:** ^1^Central Laboratory, Gansu Province Academy of Chinese Medicine, Lanzhou, Gansu 730050, China; ^2^Third Hospital Affiliated to Beijing University of Traditional Chinese Medicine, Beijing 100029, China; ^3^School of Clinical Medicine, Gansu University of Traditional Chinese Medicine, Lanzhou, Gansu 730000, China; ^4^Key Laboratory of Traditional Chinese Medicine of the Ministry of Education, Beijing University of Traditional Chinese Medicine, Beijing 100029, China

## Abstract

*Background*. Insulin resistance (IR) is the main etiology of type 2 diabetes mellitus (T2DM). It has been known that total flavonoid extracts can markedly improve the hypoglycemic symptoms caused by IR. Nevertheless, the relevant molecular mechanism remains unclarified.* Aim*. This study aimed to investigate the antihyperglycemic effects and mechanism of the total flavonoid extract from* Oxytropis falcata* Bunge.* Methods*. STZ-induced T2DM rats (*n* = 35) were divided into 5 groups: model, low-, medium-, and high-dose total flavonoids, and pioglitazone groups. Ten healthy rats were used as controls. The serum insulin and inflammatory cytokines (MCP-1, TNF-*α*, and IL-6) level was measured by ELISA. The concentration of IRS-1, p-IRS-1, PKB p-PKB, PI3Kp85, and p-PI3K in skeletal muscles was determined by Western blot. The mRNA level of GLUT4, I*κ*B, and NF-*κ*B in skeletal muscle was detected by qRT-PCR.* Results*. The treatment of medium- and high-dose total flavonoids significantly reduced the FPG and P2hPG and enhanced insulin level in T2DM rats (*P* < 0.05). When compared with controls, the serum level of MCP-1, TNF-*α*, IL-6, IRS-1, and p-IRS-1 was significantly increased in T2DM rats, but the level of PKB, p-PKB, PI3Kp85, and p-PI3K expression was reduced (*P* < 0.05). The GLUT4 and I*κ*B mRNA expression were significantly decreased, and NF-*κ*B mRNA level was increased (*P* < 0.05). The treatment of low-, medium-, or high-dose total flavonoids markedly reversed the changes above (*P* < 0.05).* Conclusion*. Our study has confirmed the therapeutic effects of total flavonoids from* Oxytropis falcata* Bunge on IR. The flavonoids might reduce the production of inflammatory cytokines through downregulation of NF-*κ*B expression in inflammatory pathway and regulate the IRS-1-PI3-K-PKB/Akt insulin pathway and thereby increased the GLUT4 expression.

## 1. Introduction

With the rapid development of global economy, the incidence of type 2 diabetes mellitus (T2DM) has substantially increased. The prevalence of T2DM in China and USA has recently reached as high as 11.6% and 11.3%, respectively [1, 2]. Insulin resistance (IR), the main etiology of T2DM, refers to the physiological condition in which cells in the body become resistant to the normal functions of insulin. Patients with IR may also develop a variety of diseases such as obesity, hyperlipidemia, cardiovascular diseases, essential hypotension, and nonalcoholic fatty liver disease [3, 4]. Studies have suggested an important role of inflammation in IR [5–7]. NF-kB (nuclear factor kappa-light-chain-enhancer of activated B cells), one of the important inflammatory pathways, can decrease the expression of insulin receptor substrate 1 (IRS1) in patients with IR [8]. A number of other inflammatory factors are also involved in IR including TNF-*α*, IL-1, IL-6, monocyte chemoattractant protein 1 (MCP-1), plasminogen activator inhibitor-1 (PAI-1), intercellular adhesion molecule 1 (ICAM-1), and vascular cell adhesion molecule 1 (VCAM-1) [9].

*Oxytropis falcata* Bunge, a species belonging to the genus* Oxytropis*, is mainly distributed in Western China, including Qinghai, Gansu, Tibet, and Sichuan. The plant has been used in folk medicine for the treatment of inflammation. Studies have found that the chloroform extract of* Oxytropis falcata* Bunge, primarily flavonoid compounds, has strong anti-inflammatory and antioxidant effect [10–12]. Several studies have also demonstrated that the total flavonoid extract can markedly improve the hypoglycemic symptoms caused by IR [13–15]. Nevertheless, the molecular mechanism behind the therapeutic effect of the total flavonoid extract on IR remains unclarified. In the current study, the antihyperglycemic effects of the total flavonoid extract from* Oxytropis falcata* Bunge were verified in streptozotocin- (STZ-) induced type 2 diabetic rats. The changes in inflammatory factors and NF-kB pathway, as well as insulin signaling pathway, were examined to explore the possible underlying mechanisms.

## 2. Material and Methods

### 2.1. Animals

Specific pathogen-free (SPF) male Wistar rats weighing (200 ± 20) g were purchased from Gansu University of Traditional Chinese Medicine (license number SCXK (Gan) 2004-0006). The rats were raised with free access to water and food in a barrier-level animal laboratory at the University for a week before the experiments. The experiment protocol was approved by the Research Ethics Committee at the University.

### 2.2. Main Reagents

*Oxytropis falcata* Bunge was purchased from Youruntang Co., Ltd. (Qinghai, China). Total flavonoid (36.58% purity) was extracted by Usea Biotech (Shanghai, China). Pioglitazone hydrochloride tablets were manufactured by Taiyang Pharmaceutical Co. (Beijing, China, lot number 130605). STZ was purchased from Sigma (St. Louis, MO, USA). The insulin ELISA detection kit and MCP-1 ELISA kit were purchased from Cusabio (Wuhan, China). ELISA kits for TNF-*α* and IL-6 were purchased from Dakewe Biotech (Shenzhen, China). Roche Accu-Chek active blood glucose test strips were purchased from Roche Diagnostics GmbH (Mannheim, Germany). Protein extraction kit and BCA kit were purchased from Beyotime Institute of Biotechnology (Shanghai, China). Rabbit anti-mouse IRS1 (ab52167, 1 : 500 dilution), PKB (ab25893, 1 : 1000 dilution), p-PKB (ab81283, 1 : 5000 dilution), and p-PI3K (ab182651, 1 : 1000 dilution) antibodies were purchased from Abcam (Cambridge, MA, USA). Rabbit anti-mouse p-IRS1 (catalog number 2381, 1 : 1000 dilution) and PI3K (catalog number 4292, 1 : 1000 dilution) were purchased from Cell Signaling (Beverly, MA, USA). TRIzol extraction kit was purchased from Invitrogen (Carlsbad, CA, USA). M-MLV reverse transcription kit and DreamTaq Green PCR Master Mix (2x) were purchased from Takara (Tokyo, Japan). Real-time PCR amplification kit was purchased from Zhongyuan Ltd. (Beijing, China). The primers were synthesized by Sangon Biotech (Shanghai, China).

### 2.3. Construction of Animal Models and Medication

Ten rats in the normal control group were given normal diet throughout the experiment. The remaining rats were given high-sugar and high-fat diet for 8 weeks, followed by an injection of STZ (25 mg·kg^−1^·d^−1^) for 1 week. Blood was collected via the tail vein, and fasting plasma glucose (FPG) and postprandial 2-hour plasma glucose (P2hPG) were measured with an Accu-Chek Active blood glucose meter (Roche Diagnostics). Rats with stable blood sugar above 13.9 mmol/L were diagnosed as T2DM. A total of 35 T2DM rats were randomly divided into 5 groups: model, low-, medium-, and high-dose total flavonoids, and pioglitazone groups (*n* = 7). Low-, medium-, and high-dose total flavonoids groups were given intragastric administration of 10 ml·kg^−1^ of 100, 200, and 400 mg·kg^−1^·d^−1^, respectively, for 4 weeks. Pioglitazone was given 10 ml·kg^−1^ of 10 mg·kg^−1^·d^−1^. Normal control and model groups were given equal volume of saline. After 4 weeks, all rats were fastened for 8 h and anesthetized. Blood sample was taken from the hearts. Rats were sacrificed and skeletal muscles were collected for subsequent analysis.

### 2.4. ELISA

The level of serum insulin and inflammatory cytokines (MCP-1, TNF-*α*, and IL-6) was measured using the ELISA kit according to manufacturer's instructions. The OD value of each sample was detected at the wavelength of 450 nm with a Bio-Rad 680 microplate reader (Bio-Rad, Hercules, CA, USA).

### 2.5. Western Blot Analysis

The concentration of IRS-1, p-IRS-1, PKB p-PKB, PI3Kp85, and p-PI3K in skeletal muscles was determined by Western blot. Briefly, tissues were homogenized in liquid nitrogen. Total protein was extracted using protein extraction kits and quantified using the BCA kit following the manufacturer's instructions. Equal amounts of total protein (20 *μ*g) were separated by SDS-PAGE electrophoresis and transferred to polyvinylidene difluoride membranes. The membrane was blocked in TBS buffer containing 5% skim milk and 0.1% Tween 20 at room temperature for 2 h and incubated with the appropriate primary antibody overnight at 4°C with gentle shaking. The HRP-labeled secondary antibodies were added and the membranes were incubated at 37°C for 1 h. The membranes were washed 3 times with TBST for 5 min each and subjected to ECL detection. The intensity of bands was detected by a Molecular Imager® ChemiDoc™ XRS System (Bio-Rad Laboratories). The gray value of bands was analyzed by Image-Pro Plus software (Bio-Rad Laboratories). The relative expression of target protein was calculated as the ratio of its gray value to that of the internal control GAPDH.

### 2.6. Quantitative Reverse Transcription PCR (qRT-PCR)

Total RNA was extracted using RNA extraction kit and quantified using a spectrometer under a wavelength of 260 nm. Total RNA was reverse-transcribed into cDNA using reverse transcription kit following the manufacturer's instructions. The mRNA level of GLUT4, I*κ*B, and NF-*κ*B in skeletal muscle was detected by qRT-PCR using cDNA as template and DreamTaq Green PCR Master Mix. The reaction condition was as follows: 94°C 10 min; 45 cycles of 94°C 15 s, 60°C 60 s, and 72°C 10 min. Primer sequences were I*κ*B forward: 5′-TGACCATGGAAGTGATTGGT-3′, reverse: 5′- AGCCAAGTGGAGTGGAGTCT-3′; NF-*κ*B forward: 5′-CGTGAGGCTGTTTGGTTTGA-3′, reverse: 5′-CTTATGGCTGAGGTCTGGTC-3′; GLUT4 forward: 5′-GTTGGTCTCGGTGCTCTTAG-3′, reverse: 5′-GGCCACGATGGACACATAAC-3′; and *β*-actin forward: 5′-GCAGTTGGTTGGAGCAA-3′, reverse: 5′-ATGCCGTGGATACTTGGA-3′. The experiment was repeated three times. Data was analyzed using the 2-ΔCt method.

### 2.7. Statistical Analysis

All data were expressed as mean ± standard deviation and analyzed using SPSS 19.0 (SPSS Inc., Chicago, IL, USA). Difference between groups was compared by ANOVA. *P* values smaller than 0.05 are considered statistically significant.

## 3. Results

### 3.1. Comparison of Serum Level of Insulin, FPG, and P2hPG

As shown in Table 1, the level of FPG and P2hPG in model group was significantly increased compared with normal controls, whereas serum insulin level was markedly decreased (*P* < 0.05). The treatment of medium- and high-dose total flavonoids, as well as pioglitazone, had significantly reduced the FPG and P2hPG and enhanced insulin level in T2DM rats (*P* < 0.05). The low-dose total flavonoids treatment had little effect on serum level of insulin, FPG, and P2hPG.

### 3.2. Comparison of Serum MCP-1, TNF-*α*, and IL-6 Concentration

The serum level of MCP-1, TNF-*α*, and IL-6 in T2DM rats was significantly increased (*P* < 0.05) but was substantially reduced after pioglitazone treatment. While both medium- and high-dose total flavonoids significantly decreased the level of IL-6, only medium-dose treatment reduced the production of MCP-1 (*P* < 0.05). None of the total flavonoids group affected the TNF-*α* level (Table 2).

### 3.3. Comparison of the Expression of Insulin Signaling Molecules in Skeletal Muscles

**Figure 1:**
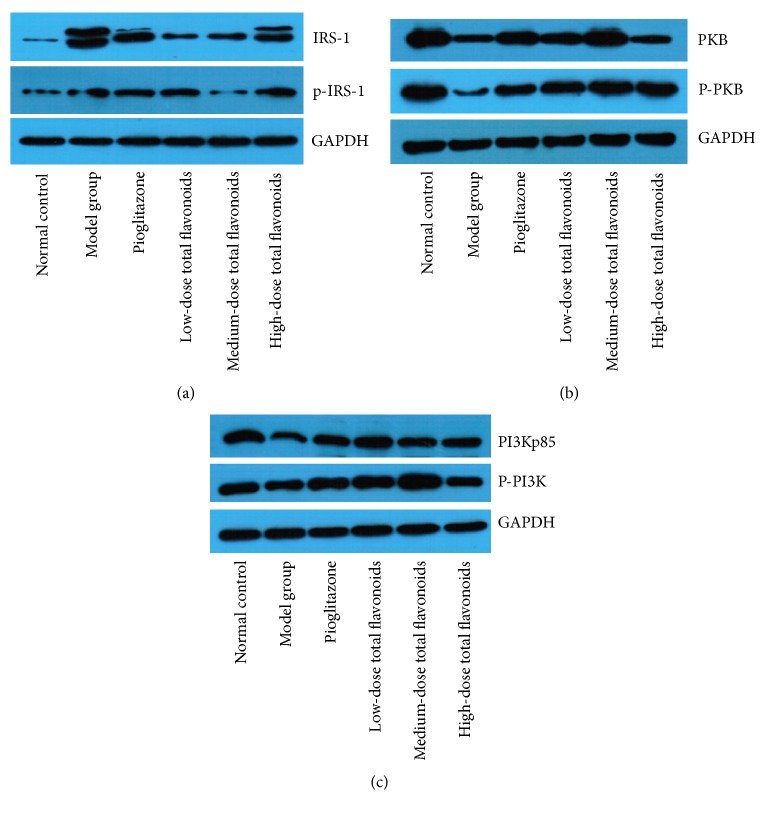
Western blot analysis of the expression of insulin signaling molecules in skeletal muscles in different groups (a) IRS-1 and p-IRS-1; (b) PKB and p-PKB; and (c) PI3Kp85 and p-PI3Kp85. (1) Normal control, (2) model group, (3) pioglitazone, (4) low-dose total flavonoids, (5) medium-dose total flavonoids, and (6) high-dose total flavonoids.

The relative expression of proteins involved in the insulin signaling pathway was examined by Western blot including IRS-1, PKB, and PI3Kp85 (Table 3, Figures 1(a)–1(c)). The expression of IRS-1 and p-IRS-1 in model group was significantly higher than that in normal controls (*P* < 0.05). The IRS-1 level in low-, medium-, and high-dose total flavonoids groups was significantly reduced (*P* < 0.05). The p-IRS-1 expression in pioglitazone group was also decreased (*P* < 0.05). The expression of PKB, p-PKB, PI3Kp85, and p-PI3K in model group was significantly decreased compared with normal control group (*P* < 0.05). The p-PKB level in all treatment groups was markedly higher than that in model group (*P* < 0.05). The PKB and p-PI3K expression in pioglitazone and low- and medium-dose total flavonoids groups were significantly increased (*P* < 0.05). The expression of PI3Kp85 in pioglitazone and medium- and high-dose total flavonoids groups was also increased (*P* < 0.05).

### 3.4. Comparison of GLUT4, I*κ*B, and NF-*κ*B mRNA Expression in Skeletal Muscles

As shown in Table 4, the GLUT4 and I*κ*B mRNA in model group were significantly reduced compared with normal controls, whereas the NF-*κ*B mRNA was significantly increased (*P* < 0.05). When compared with the model group, GLUT4 mRNA in pioglitazone and the three total flavonoids groups was markedly enhanced, and NF-*κ*B mRNA expression was reduced (*P* < 0.05).

## 4. Discussion

Diabetes is one of the most common chronic diseases with increasingly higher prevalence. The pathogenesis of diabetes is a complex process involving many factors. T2DM has been known to be induced by IR. Therefore, investigation on improving IR is of significant clinical importance for the prevention and treatment of T2DM.

In most T2DM patients, insulin postreceptor signaling transduction disorder in target cells is the main mechanism leading to IR [16]. Numerous studies have suggested that IR is a chronic nonspecific inflammation involving inflammatory cytokines, immune system, and adipose tissues [17–20]. Inflammation has interfered the insulin postreceptor signaling pathway, IRS-1-PI3-K-PKB/Akt pathway, and thereby induces the RI [21, 22]. Specifically, inflammation can induce the serine/threonine phosphorylation of IRS-1, which affects the interaction between IRS-1 and insulin receptor, and inhibits the normal tyrosine phosphorylation of IRS-1. The reduced IRS-1 activity leads to reduced phosphorylation of PI3-K and thereby decreased expression of GLUT4 in skeletal muscle cells and adipocytes. Consequently, the biological activity of insulin was reduced, resulting in the occurrence of IR. IKK*β*/NF-*κ*B is one of the most frequently studied inflammatory signaling pathways associated with inflammation-induced IR. Under normal conditions, the I*κ*B protein (inhibitor of NF-*κ*B) binds to NF-*κ*B in the cytoplasm, whereas IKK*β*, the kinase of I*κ*B, is activated by the stimulation of inflammatory cytokines such as TNF-*α*, IL-1, and IL-6 during inflammation, leading to the serine phosphorylation of I*κ*B. The NF-*κ*B is translocated into the nucleus, where it binds to the genomic DNA and regulates the expression of inflammatory cytokines, initiating or aggregating the inflammatory response and ultimately leading to the occurrence of IR. Studies have suggested that the IR can be prevented by the inhibition of the IKK*β*/NF-*κ*B pathway [23]. In this study, an IR rat model was successfully constructed by high-fat high-sugar diet and injection of STZ, as indicated by increased blood FPG and P2hPG and decreased serum insulin level. We detected unusually high level of inflammatory cytokines (MCP-1, TNF-*α*, and IL-6) in the serum of these IR rats. Moreover, these rats developed significantly enhanced IRS-1, p-IRS-1, and NF-*κ*B, as well as reduced PI3Kp85, p-PI3K, PKB, p-PKB, GLUT4, and I*κ*B level in the skeletal muscles. These results suggested a close association between the inflammatory pathway and the IRS-1-PI3-K-PKB/Akt insulin postreceptor pathway, which is consistent with previous studies.

Currently, the drawbacks of Western medicine in the treatment of IR have been frequently reported including adverse side effects and drug resistance. Therefore, exploration of herbal medicines for IR has recently become a research hotspot due to their high safety. It has been found that the flavonoids compounds in several plants such as buckwheat leaf, corn silk, and kudzu root have hypoglycemic effects [24–26], suggesting the potential of flavonoids compounds as effective and safe medicines for the treatment of IR and diabetes. Consistently, in our study, the treatment with medium- and high-dose total flavonoids from* Oxytropis falcata* Bunge significantly reduced the blood FPG and P2hPG and stimulated the secretion of insulin in IR rats, suggesting the therapeutic effects of the total flavonoids.

Nevertheless, the molecular mechanism underlying the hypoglycemic effects of total flavonoids has not been well clarified. Studies have found that the total flavonoids from* Oxytropis falcata* Bunge are mainly composed of flavonoid glycoside, flavanone, flavonols, chalcone, dihydrochalcone, flavanones, and isoflavane and have strong anti-inflammatory and antioxidant effects [27]. In this study, we monitored the changes in the IRS-1-PI3-K-PKB/Akt insulin postreceptor pathway and inflammatory pathway after the treatment of* Oxytropis falcata* Bunge total flavonoids. It was found that* Oxytropis falcata* Bunge total flavonoids significantly reduced the level of MCP-1 and IL-6 when compared with model group. The flavonoids compounds also inhibited the expression of IRS-1, p-IRS-1, PKB, p-PKB, PI3Kp85, and p-PI3K. Moreover, the flavonoids markedly stimulated the expression of GLUT4 and reduced NF-*κ*B in the treatment groups.

Overall, our study has confirmed the therapeutic effects of total flavonoids extracted from* Oxytropis falcata* Bunge on IR. The flavonoids might reduce the production of inflammatory cytokines through downregulation of NF-*κ*B expression in inflammatory pathway and regulate the IRS-1-PI3-K-PKB/Akt insulin pathway and thereby increased the GLUT4 expression. Consequently, the glucose uptake and utilization in skeletal muscles are improved, and glucose balance is recovered. Our study has provided insights into the therapeutic mechanism of* Oxytropis falcata* Bunge total flavonoids on IR, which will be a basis for the development of effective and safe natural medicine for the treatment of T2DM.

## Figures and Tables

**Table 1:** Comparison of *serum level of FPG, P2hPG*, and insulin in difference groups (*n* = 7, $\overset{-}{x}\pm s$).

Group	Dosage (mg·kg^−1^)	FPG (mmol/l)	P2hPG (mmol/l)	Insulin (nIU/ml)
Normal control	—	4.30 ± 0.20	6.06 ± 0.44	90.04 ± 18.73
Model	—	25.34 ± 3.88^(1)^	28.90 ± 3.38^(1)^	19.42 ± 19.36^(1)^
Pioglitazone	10	11.80 ± 1.04^(2)^	14.74 ± 2.26^(2)^	43.44 ± 57.10^(2)^
Low-dose total flavonoids	100	22.86 ± 4.64	25.69 ± 4.34	36.52 ± 33.85
Medium-dose total flavonoids	200	16.09 ± 2.25^(2)^	19.89 ± 1.91^(2)^	47.40 ± 34.77^(2)^
High-dose total flavonoids	400	13.13 ± 0.80^(2)^	14.34 ± 2.12^(2)^	49.60 ± 47.60^(2)^

^(1)^ *P* < 0.05 compared with normal control group and ^(2)^*P* < 0.05 compared with model group.

**Table 2:** Comparison of serum level of MCP-1, TNF-*α*, and IL-6 in different groups (*n* = 7, $\overset{-}{x}\pm s$).

Group	Dosage (mg·kg^−1^)	MCP-1 (pg/ml)	TNF-*α* (pg/ml)	IL-6 (pg/ml)
Normal control	—	88.83 ± 23.69	104.44 ± 10.84	65.44 ± 5.61
Model	—	172.03 ± 78.84^(1)^	131.92 ± 44.13^(1)^	120.30 ± 102.16^(1)^
Pioglitazone	10	111.34 ± 26.25^(2)^	109.29 ± 14.90^(2)^	83.58 ± 44.60^(2)^
Low-dose total flavonoids	100	156.19 ± 92.01	120.53 ± 27.42	92.93 ± 25.60^(2)^
Medium-dose total flavonoids	200	101.84 ± 31.71^(2)^	116.62 ± 15.56	68.84 ± 20.76^(2)^
High-dose total flavonoids	400	133.54 ± 51.55	114.67 ± 21.28	48.68 ± 32.57^(2)^

^(1)^ *P* < 0.05 compared with normal control group and ^(2)^*P* < 0.05 compared with model group.

**Table 3:** Comparison of the expression of insulin signaling molecules in skeletal muscles in different groups (*n* = 3, $\overset{-}{x}\pm s$).

Group	Dosage (mg·kg^−1^)	IRS-1	p-IRS-1	PKB	p-PKB	PI3Kp85	p- PI3K
Normal control	—	0.12 ± 0.06	0.22 ± 0.08	1.90 ± 0.80	1.52 ± 0.39	1.56 ± 0.79	1.38 ± 0.24
Model	—	0.99 ± 0.44^(1)^	0.75 ± 0.09^(1)^	0.76 ± 0.33^(1)^	0.40 ± 0.04^(1)^	0.59 ± 0.34^(1)^	0.52 ± 0.11^(1)^
Pioglitazone	10	0.87 ± 0.36	0.43 ± 0.28^(2)^	1.36 ± 0.50^(2)^	0.93 ± 0.24^(2)^	0.90 ± 0.55^(2)^	1.01 ± 0.10^(2)^
Low-dose total flavonoids	100	0.36 ± 0.12^(2)^	0.55 ± 0.35	1.01 ± 0.48^(2)^	1.01 ± 0.40^(2)^	0.78 ± 0.41	1.50 ± 0.16^(2)^
Medium-dose total flavonoids	200	0.36 ± 0.10^(2)^	0.56 ± 0.18	1.66 ± 0.57^(2)^	1.14 ± 0.50^(2)^	0.93 ± 0.23^(2)^	1.10 ± 0.38^(2)^
High-dose total flavonoids	400	0.44 ± 0.32^(2)^	0.42 ± 0.21^(2)^	0.82 ± 0.11	1.42 ± 0.09^(2)^	1.07 ± 0.24^(2)^	0.88 ± 0.21

^(1)^ *P* < 0.05 compared with normal control group and ^(2)^*P* < 0.05 compared with model group.

**Table 4:** Comparison of the mRNA expression of GLUT4, I*κ*B, and NF-*κ*B in skeletal muscles in different groups (*n* = 3, $\overset{-}{x}\pm s$).

Group	Dosage (mg·kg^−1^)	GLUT4	I*κ*B	NF-*κ*B
Normal control	—	1.351 ± 1.18	1.003 ± 0.09	1.253 ± 0.79
Model	—	0.098 ± 0.04^(1)^	0.539 ± 0.11^(1)^	8.881 ± 4.46^(1)^
Pioglitazone	10	0.674 ± 0.14^(2)^	0.842 ± 0.78	3.609 ± 1.69^(2)^
Low-dose total flavonoids	100	0.182 ± 0.06^(2)^	0.526 ± 0.33	3.968 ± 1.45^(2)^
Medium-dose total flavonoids	200	0.355 ± 0.05^(2)^	0.848 ± 0.57	3.621 ± 0.65^(2)^
High-dose total flavonoids	400	0.234 ± 0.08^(2)^	0.867 ± 0.62	2.695 ± 0.70^(2)^

^(1)^ *P* < 0.05 compared with normal control group and ^(2)^*P* < 0.05 compared with model group.
